# Functional and Structural Characterization of PETase SM14 from Marine-Sponge Streptomyces sp. Active on Polyethylene Terephthalate

**DOI:** 10.1021/acssuschemeng.5c00737

**Published:** 2025-05-15

**Authors:** Alan Carletti, Shapla Bhattacharya, Sara Pedroni, Marcello Berto, Riccardo Bonettini, Rossella Castagna, Emilio Parisini, Giulia Di Rocco

**Affiliations:** † Department of Life Sciences, University of Modena and Reggio Emilia, Via Campi 103, 41125 Modena, Italy; ‡ Department of Biotechnology, 187008 Latvian Institute of Organic Synthesis, Aizkraukles 21, LV-1006 Riga, Latvia; § Faculty of Materials Science and Applied Chemistry, Riga Technical University, Paula Valdena 3, LV-1048 Riga, Latvia; ∥ Department of Chemistry “G. Ciamician”, University of Bologna, Via P. Gobetti 85, 40129 Bologna, Italy

**Keywords:** polyethylene terephthalate, PETase, X-ray structure, salt tolerance, plastic waste recovery

## Abstract

The recent discovery of the PETase enzyme family offers a sustainable solution for depolymerizing poly­(ethylene terephthalate) (PET), one of the most widespread plastic compounds, under mild conditions. This enables the environmentally beneficial conversion of plastic waste into value-added products. Among this enzyme family, PETase from Ideonella sakaiensis has been the most extensively studied. Although other similar enzymes have been discovered, our knowledge about the catalytic and structural properties of this class remains limited. In this study, a PETase-like enzyme (PETase SM14) from Streptomyces sp. SM14 was heterologously produced in Escherichia coli, and its activity was tested on post-consumer plastic substrates using high-performance liquid chromatography for product quantification as well as scanning electron microscopy and atomic force microscopy for substrate surface imaging evaluation. PETase SM14 exhibited high salt tolerance (1.5 M), good heat resistance (Tm 56.26 °C), and optimal activity at pH 9.0, highlighting its potential for PET waste bioremediation. Furthermore, its X-ray crystal structure was solved at 1.43 Å resolution, revealing conserved features of the PETase family with potential relevance for future engineering applications.

## Graphical abstract

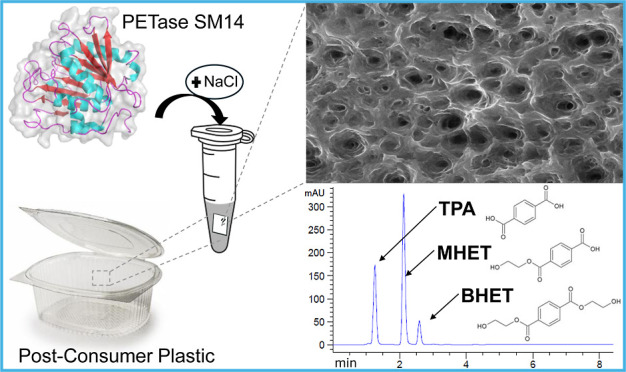

## Introduction

Plastic materials, known for being cost-effective, versatile, and durable, have seen rapid growth in global production, with 8.3 million tons produced between 1950 and 2015.^1,2^ However, inadequate recycling and limited circular reuse have resulted in significant waste accumulation, raising serious environmental concerns due to plastics’ resistance to natural degradation processes.^3^ Polyethylene terephthalate (PET) is widely used, inherently stable, and environmentally persistent.^4^ Although PET is technically recyclable, only a small fraction is currently processed through recycling efforts, while a majority of PET waste persists in natural habitats. The global PET market is projected to reach USD 109 billion by 2032, with a significant annual growth rate (9.5%),^5^ highlighting an urgent need for sustainable management and recycling solutions, especially in developing countries, where advanced recycling infrastructures and regulatory frameworks are lacking.^6^ While traditional PET recycling methods such as thermo-mechanical and chemical processes have been developed,^7^ the efficiency remains limited, and there is a pressing demand for alternative approaches. One promising avenue lies in the discovery of microbial enzymes capable of degrading PET. Recent studies have identified enzymes—including members of the hydrolase family—cutinases (EC 3.1.1.74), lipases (EC 3.1.1.3), and carboxylesterases (EC 3.1.1.1/EC 3.1.1.101/EC 3.1.1.2)—that exhibit activity on low-crystallinity, low-density polymers of PET and related compounds. These microbial enzymes show varied structural adaptations that enhance their efficacy under distinct conditions. Bacterial PET-degrading enzymes, such as *Is*PETase,^8^ have been categorized into types I and II based on structural characteristics; type II enzymes, in particular, feature modifications like additional disulfide bonds and extended loops near their active sites, enhancing substrate accessibility.^9^ Despite over 20 years of research on the enzymatic degradation of PET, microbial enzymes still exhibit relatively low turnover rates, reflecting the unique challenges presented by PET as a non-natural substrate.^10^ To date, 119 wild-type PET-active enzymes have been characterized, with 35 enzymes having their 3D structures resolved.^11^ This growing body of knowledge offers a foundation for advancing enzyme-based approaches to PET recycling, holding promise for more sustainable and efficient plastic waste management solutions in the future. In recent years, concerted efforts have been made to characterize each aspect of these enzymes^12–15^ and enhance their performance through molecular engineering, which often resulted in the creation of mutants featuring greater thermal stability and increased catalytic properties.^16–19^ Another emerging area of interest involves the influence of specific salts on the properties of these enzymes, particularly, their catalytic activity and thermostability. For instance, Ca^2+^ has been shown to alter the tertiary structure of PETases, leading to increased activity and thermal stability.^20,21^ Similarly, K^+^ and Na^+^ ions may exert effects analogous to Ca^2+^, as evidenced in a recent study.^22^ Additionally, Schmidt et al.^23^ demonstrated that the activity of the polyester hydrolases LCC and TfCut2 on PET films strongly depends on the type and concentration of the buffer. High initial hydrolysis rates were observed using sodium phosphate buffer concentrations exceeding 0.7 M, emphasizing the role of specific salts in modulating enzyme performance.

In this work, we describe a candidate PETase-like enzyme, SM14 (PETase SM14 hereafter), isolated from the marine-sponge Streptomyces sp. SM14.^24^ The enzyme was heterologously expressed in Escherichia coli to characterize its polyesterase activity. This was confirmed using a PET-based post-consumer plastic (PCP) through liquid chromatography assays (HPLC) and scanning electron microscopy (SEM) analysis. Results revealed that the presence of NaCl was essential for enzymatic catalysis, corroborating the enzyme’s halophilic nature and superior salt tolerance compared to *Is*PETase, which exhibits optimal activity at low salt concentrations. Further X-ray crystallographic analysis of PETase SM14 revealed its three-dimensional structure, which was compared to the well-characterized *Is*PETase and with a polyester hydrolase (PE-H) from Pseudomonas aestusnigri, another marine bacterium. All three enzymes share conserved motifs, including the serine hydrolase sequence Gly-x1-Ser-x2-Gly and the catalytic triad Ser-Asp-His. These findings enhance our understanding of PETase-like enzymes and their potential applications in PET degradation.

## Results and Discussion

### Production of PETase SM14

The protein was expressed in E. coli BL21 (DE3) and purified as indicated in the Materials and Methods section. The procedure led to the production of 162 mg of pure protein from 4.5 g of cellular pellet, resulting in a yield of 3.6%. The identity of the protein was confirmed by peptide-mass fingerprinting. Using the MASCOT search database, the detected peptide sequences were compared against all sequences stored in an in-house database as well as the SwissProt database. This analysis revealed a match with the mature form of PETase SM14. Notably, the sample achieved a 95% sequence coverage (Figure S1). Circular dichroism (CD) data (Figure S2) confirm proper protein folding and align with the structure determined by X-ray crystallography. The melting temperature (Tm) of the enzyme was determined to be 56.26 °C (Figure S2) by CD spectroscopy. To evaluate the enzyme’s stability at varying pH values and NaCl concentrations, its melting temperature (Tm) was determined using a thermal shift assay across a pH range of 6.0–9.0 with NaCl concentrations from 100 to 700 mM (Table S1). Changes in buffer pH had a minimal impact on Tm, which remained relatively constant. Increasing the salt concentration up to 700 mM in buffer Tris pH 8.0 did not result in unfolding of the protein, as shown by the non-decreasing Tm [°C]. This finding confirms the enzyme’s stability at higher salt concentrations (Figure S3).

### Structural Features

The X-ray structure of PETase SM14 (UniProt ID: A0A679PDB4) validated the accuracy of the predicted AlphaFold structure, exhibiting a high structural identity with a root-mean-square deviation (RMSD) of 0.278 Å. Notably, this extends to the loop regions, where the catalytic site is located. *Is*PETase, a well-known enzyme active on PET,^15,25^ has been extensively characterized.^14,26^ In this study, *Is*PETase and a polyester hydrolase (PE-H) identified in the genome of the marine hydrocarbonoclastic bacterium P. aestusnigri^27^ were employed for comparative structural analysis of PETase SM14. Structural alignment of the three enzymes shows a high degree of similarity, with an RMSD of 0.691 Å across 190 Cα atoms for *Is*PETase and an RMSD of 0.81 Å over 195 Cα atoms for PE-H including the catalytic triad within the active site pocket (Figure 1). This indicates that these enzymes degrade PET through the same catalytic mechanism. The serine-hydrolase mechanism involves three conserved residues: serine, histidine, and aspartate, which retain their positions and orientation within the structures of the two enzymes.

**Figure 1:**
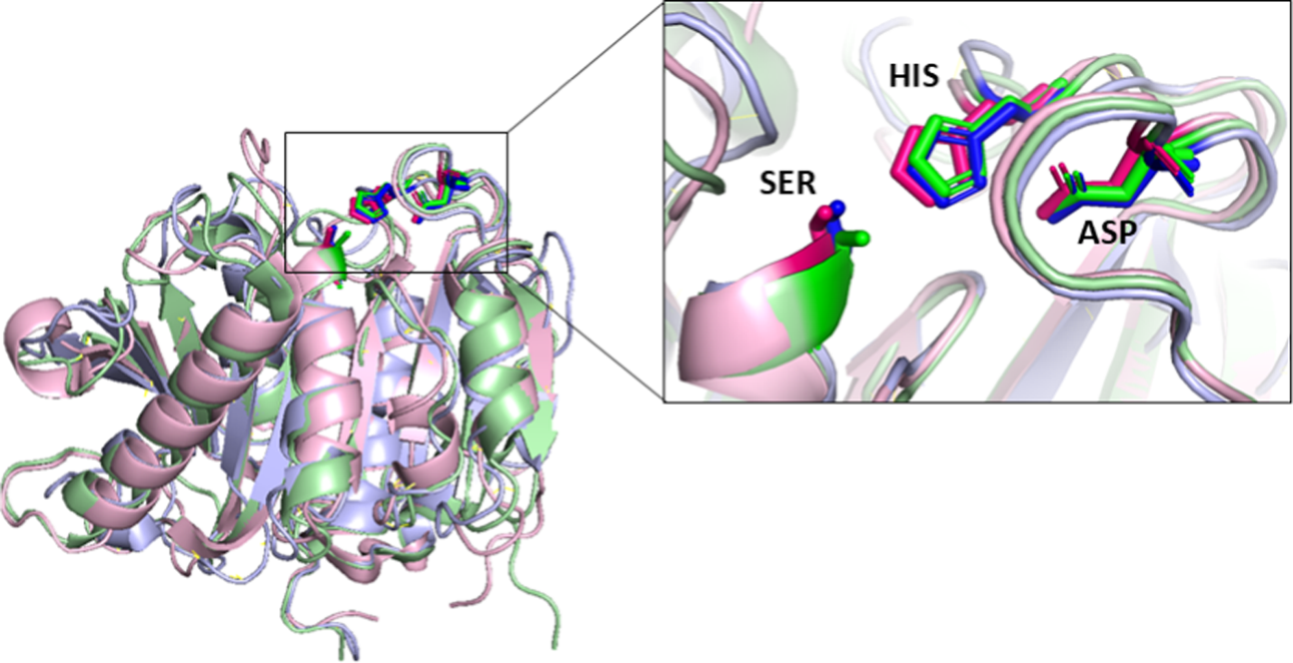
Overall structure comparison of PETase SM14 (light blue), *Is*PETase (PDB code 6ILW, light green), and PE-H from P. aestusnigri (PDB code: 6SBN, light pink) performed using PyMOL. The structures are displayed as cartoon models, with the catalytic triad regions highlighted as stick representations in the top right inset. The catalytic triad of PETase SM14 (blue sticks) comprises S156, D202, and H234, while that of *Is*PETase (green sticks) includes S160, D206, and H237 and S171, D217, and H249 for PE-H from P. aestusnigri (pink sticks).

The electrostatic surface potentials of PETase SM14, *Is*PETase, and PE-H are shown in Figure S4. *Is*PETase features a highly polarized surface charge, resulting in an isoelectric point (pI) of 9.41, whereas PETase SM14 and PE-H have a theoretical pI of 6.67 and 6.54, respectively (as determined by the ExPASy, ProtParam tool), and both exhibit a less polarized and more delocalized surface charge. This variation in surface charges seems to affect also the active site pocket; PETase SM14 and PE-H show a slightly negative potential around the active site (red regions in Figure S4A,B), in contrast with the more positively charged pocket in *Is*PETase (Figure S4C).

The three structures primarily differ in five loop regions. While the catalytic residues align across all three, the catalytic pocket appears more closed in PE-H and SM14 compared to that in *Is*PETase. In *Is*PETase, Y87 is positioned farther from the catalytic residues, creating a more open catalytic pocket. Additionally, in the PE-H enzyme, the P96-G97-F98-V99-S99-A100-E101 sequence forms an elongated and flexible loop, which may hinder substrate interaction with the catalytic residues, potentially contributing to lower activity. The catalytic site of *Is*PETase is surrounded by conserved hydrophobic residues involved in substrate binding, including Y87, W159, M161, W185, and I208 ^14^ (Figure 2D). Structural alignment enabled the identification of the corresponding residues of PETase SM14 and PE-H, which have a nearly identical arrangement: Y88, H155, M157, W181, I204 and F98, W170, M172, W195, and I219 (Figure 2D). Hence, the catalytic site of the three enzymes exhibits a similar spatial arrangement of the aromatic and apolar residues involved in substrate binding, although some differences are present. For instance, Y88 and F98 in PETase SM14 and PE-H, respectively, are positioned in such a way that the catalytic pocket appears to be more closed (Figure 2A,B). In contrast, *Is*PETase Y87 is closer to M161, creating a more open catalytic pocket (Figure 2C). A second notable difference lies in the conserved serine-hydrolase motif Gly-x1-Ser-x2-Gly motif. In *Is*PETase, this motif consists of G158-W159-S160-M161-G162 located at the active site, typical of other enzymes in the α/β hydrolase family.^26^ In PETase SM14, however, the motif includes a histidine (H155), instead of a tryptophan (W159), which is commonly found in *Is*PETase and related hydrolases,^14,25,26^ also in PE-H (W170). Despite this substitution, the overall spatial arrangement remains analogous for both enzymes, preserving their functional capabilities.

**Figure 2:**
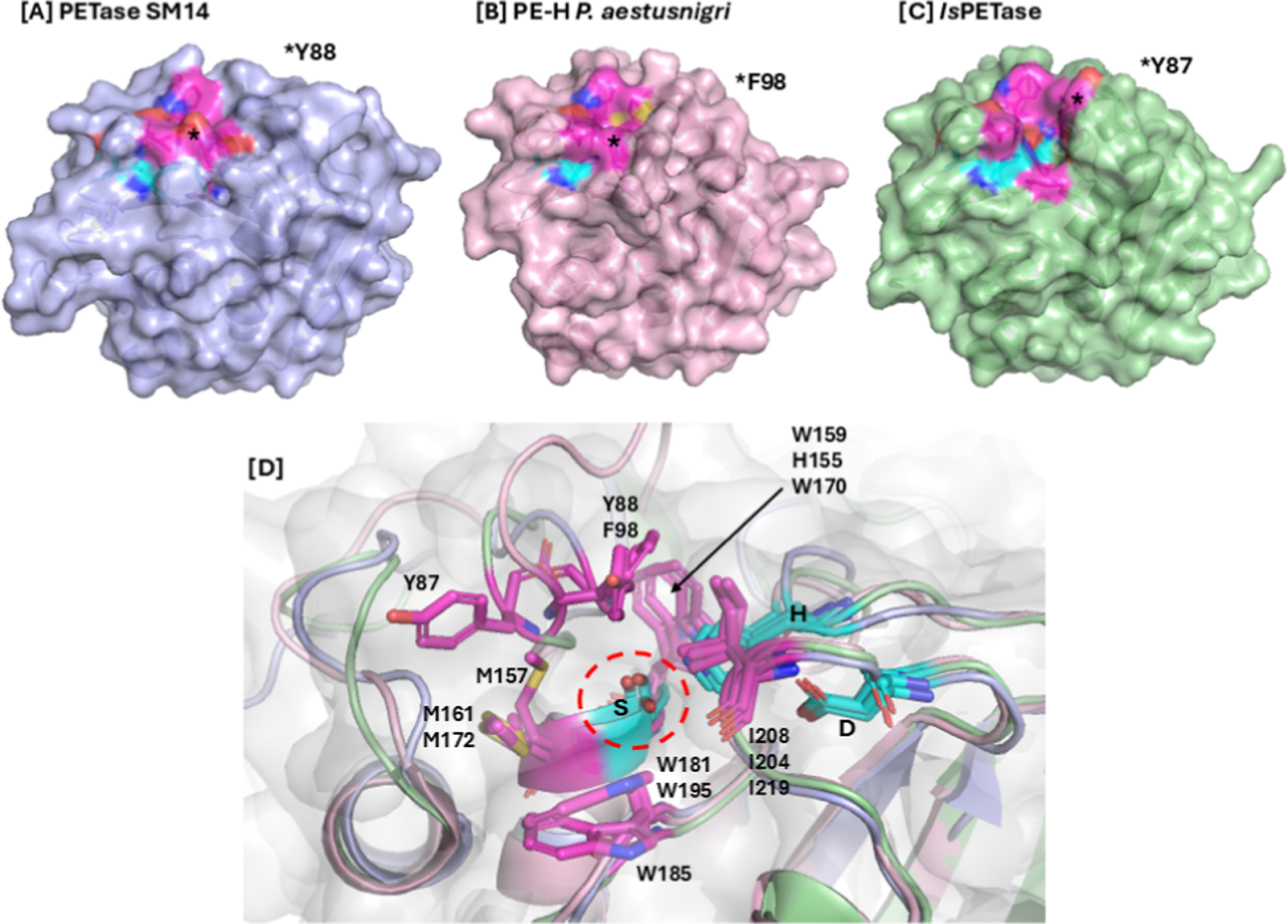
Surface representations of PETase SM14 (A), PE-H (B), and *Is*PETase (C) highlight the catalytic triad (light blue) and the putative binding site (violet). (D) Magnified view of the active site after structural alignment (cartoon model), showing the catalytic triad as light blue sticks (S156–D202–H234 of PETase SM14, S171–D217–H249 of PE-H, and S160–D206–H237 of *Is*PETase) and the putative binding site as violet sticks (Y88–H155–M157–W181–I204 of PETase SM14, F98–W170–M172–W195–I219 of PE-H, and Y87–W159–M161–W185–I208 of *Is*PETase).

### Catalytic Activity

PETase SM14 successfully degraded PCP samples, confirming the enzyme’s active form. Calibration curves of the HPLC assay method for terephthalic acid (TPA) and bis­(2-hydroxyethyl) terephthalate (BHET) were created by using standard solutions. The relative standard deviation was calculated for three repeated runs, resulting in a good linear relationship with *r* ^2^ = 0.994 for TPA and *r* ^2^ = 0.982 for BHET (Figure S5). These linear regression parameters were then utilized to quantify all of the samples.

#### Temperature and pH Dependence

The pH dependence of enzymatic activity was analyzed across a range of pH values from 6.0 to 9.0, without added salt. After 72 h of incubation, the products were analyzed using reversed-phase high-performance liquid chromatography (HPLC). The results (Figure 3) show that the TPA production is strongly pH-dependent, with a 10-fold increase in TPA production at pH 9.0 compared to pH 7.0 (≅0.02 mM TPA at pH 7.0 versus ≅0.2 mM at pH 9.0). The highest concentration of released TPA was 0.187 mM at pH 9.0 and 40 °C (Figure 3), consistent with findings for *Is*PETase produced in the chloroplasts of Chlamydomonas reinhardtii,^28^ which released TPA at a concentration of 0.191 mM under the same conditions (data not shown). The reactions conducted without the enzyme or substrate yielded negligible amounts of TPA in all measurements, confirming that PETase SM14 is responsible for product formation. The pH values of the reaction solutions remained unchanged throughout the entire reaction time.

**Figure 3:**
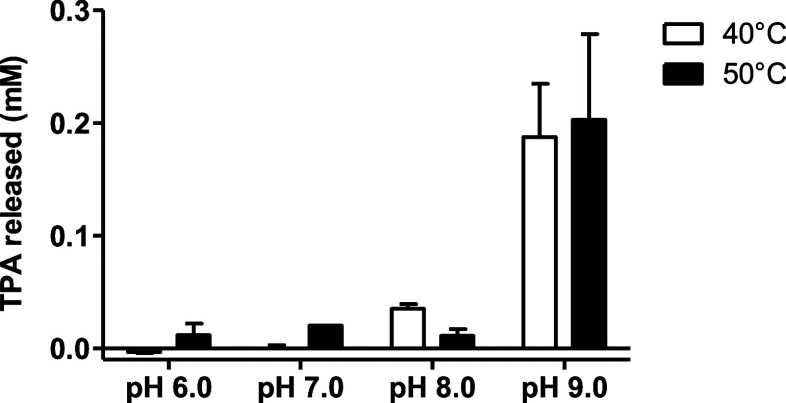
TPA production resulting from enzymatic degradation of PCP at different pH values (6.0, 7.0, 8.0, and 9.0) and temperatures (40 °C (white), 50 °C (black)) after 72 h. The black bars represent the data collected at 50 °C, while the white bars describe the data at 40 °C. The error bars show the standard deviation of the dataset relative to the mean.

Two temperatures, 50 °C and 40 °C, were tested, yielding very similar data. The optimal pH 9.0 is independent of the temperature, as the TPA released at pH 9.0 is consistent at the two temperatures (Figure 3). The glass transition temperature (Tg) of PET, defined as the temperature at which the polymer chain gain enhanced mobility, is approximately 80 °C. However, during the enzymatic hydrolysis, the Tg value of PET decreases to about 65 °C due to water molecules infiltrating the polymer chains.^29^ While *Is*PETase activity naturally decreases above 40 °C,^30^ PETase SM14 maintained structural stability up to 50 °C (Figure S2). The similar enzyme behavior at both temperatures suggests that under these reaction conditions, temperature has little effect on activity.

Within the residues of the canonical Ser-His-Asp triad, aspartate is negatively charged, while histidine and serine are partially ionized during catalysis. Notably, histidine must accept a proton from serine, creating a catalytic environment with a neutral or slightly basic optimal pH. However, the ideal pH depends on the electrostatic environment and on the structure of the active site in the presence of the substrate.^14,31,32^ Variations of the pH of the medium result in changes in the ionic state of the active site, impacting the reaction mechanism and, consequently, TPA production.^33^ For instance, histidine residues contain one or two nitrogen-bonded proton(s) in their imidazole ring with a theoretical pKa of 6.5. However, studies have determined pKa ranging from 4.7 (or even 4) to 7.5, depending on the presence of positively charged residues within the LCC esterase cavity.^34,35^ Another study calculated a pKa ≈ 10 due to the formation of a negatively charged complex.^34^ Overall, achieving the best reaction conditions at basic pH appears to be a common feature of serine esterases.^33,36^ Furthermore, the hydrophilicity of PET is accelerated by the partial alkaline hydrolysis reaction, which influences the mechanism and the dissolution rate of the reaction products, leading to the degradation of the PET outer surface.^37^

#### NaCl Dependence

The effect of environmental conditions on the PET-hydrolyzing activity of wild-type PETase SM14 was evaluated under halophilic conditions by supplementing sodium chloride in the reaction mixture (300–1500 mM). Figure 4 summarizes the results for all of the experiments conducted in the presence of NaCl at pH 9.0. Additional measurements at salt concentrations of 700 and 900 mM were performed at pH 5.0, 6.0, and 7.0; however, TPA release was negligible in all cases (data not shown). These findings indicate that a significant increase in enzyme activity due to salt concentration occurs only at pH 9.0. The concentration of TPA released as a function of salt addition is shown in Figure 4B. A notable increase in product formation is observed in the presence of salt, exceeding a 100-fold increase compared with the data obtained without salt (TPA released: 0.2 mM). From the graph in Figure 4B, two key transitions can be observed: the first one at the initial increase from no salt to a 300 mM solution and the second one between 700 and 900 mM. Thereafter, stabilization occurs, with product release remaining consistent at approximately 30 mM, even at higher salt concentrations. The PCP used in these experiments is PET-made of food packaging. The polymer matrix of this material is less homogeneous than that of a pure PET film and may exhibit regions of varied crystallinity. This contributes to the slight variability of the results, as indicated by the error bars representing the standard deviation in Figure 4B, even after four replicate analyses under the same conditions. Nevertheless, 900 mM NaCl clearly emerges as the optimal concentration for this enzyme. Morphological changes on the surfaces of PCP fragments, as examined by SEM (Figure 4D–F) after incubation with the enzyme, further confirm that 900 mM NaCl is the most effective concentration for PET degradation. The results of semi-contact atomic force microscopy (AFM) imaging in air are reported in Figure 4C, showing the effects of the protein on the morphology of the PCP sample. In particular, the samples incubated with the protein exhibit a dense network of holes on their surfaces, whereas no such features are present on the surface of the control sample (Figure S6). From AFM analysis, two parameters related to enzyme activity can be extracted: the average surface roughness of the samples σ_rms_ and the lateral correlation length ξ [Figure 5].^28,38,39^ The average values of ξ were obtained from four images of each sample. The variation in ξ, which estimates the average dimensions of the holes on the surface, can be ascribed solely to the activity of the enzyme as no other sources of degradation were present during the experimental procedure. In the control sample, ξ is related to the microscopic structure of the polymeric film. PET film samples showed a two-fold increase in ξ, from 200 ± 9 nm (control sample) to 515 ± 44 nm (after enzyme incubation) (Figure 5A). Also, σ_rms_ significantly increased from 11 ± 6 nm in the control sample to 98 ± 6 nm following the protein activity (Figure 5B). Also in this case, the increase in σ_rms_ correlates with the presence of holes in the samples incubated with the enzyme. AFM analysis further allowed for the calculation of the average depth of the holes, which was determined to be approximately 340 ± 110 nm.

**Figure 4:**
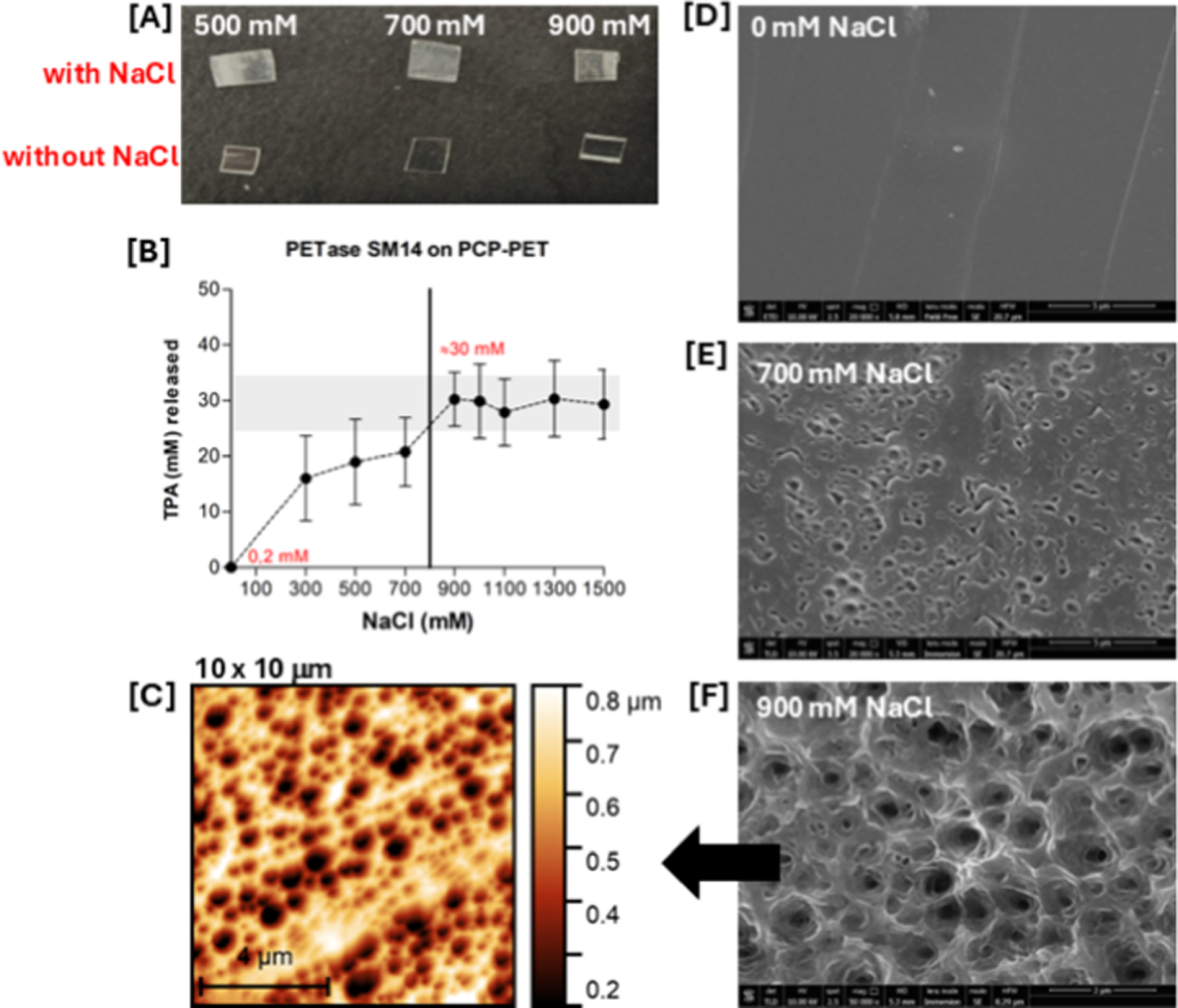
(A) Post-consumer plastic (PCP) pieces after one-week incubation with PETase under varying salinity conditions. (B) TPA release profile depending on salt concentration. Product release over 1 week (168 h) of incubation with PETase SM14 (1 μM) on PET PCP in 100 mM Tris–HCl buffer, pH 9.0, 40 °C, with sodium chloride concentrations ranging from 0 to 1500 mM. Quantification (*n* = 4) was done using the TPA standard curve. (C) AFM topographical images (10 μm × 10 μm) of PCP sample incubated with PETase SM14 and 0.9 M NaCl. (D–F) SEM micrographs of the PET PCP piece without salts (D), with 700 mM NaCl (E) and with 900 mM NaCl (F). All experiments were conducted in triplicate.

**Figure 5:**
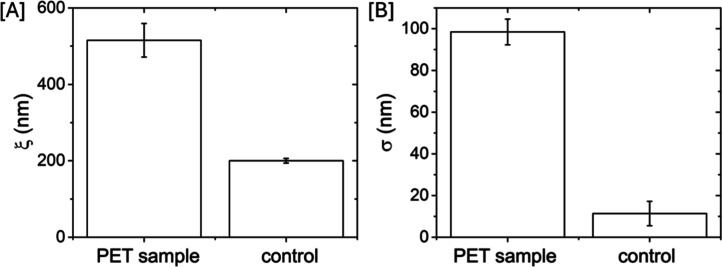
Morphological parameters extracted from AFM analysis. (A) Variation in correlation length (ξ) and (B) variation in roughness (σ_rms_) between PET samples incubated with PETase SM14 and those incubated in the control solution, i.e., in the absence of the enzyme (Figure S6). Standard deviations, calculated from four images of each sample, are reported as errors bars.

Several factors influence polymer biodegradation rates, including crystallinity, chemical properties, and molecular weight (MW). PET polymers have a crystallinity of 30–50%, an average MW of 25,000, and are hydrophobic due to chain packing,^40^ even though PET’s backbone contains ester bonds and polar functionalities such as carboxyl and hydroxyl groups that provide polarity to the polymer. The numerous benzene rings in the PET chain produce π–π interactions that enhance dipole connections and encourage the polymer’s crystalline organization by orienting the rings.^41^ Positive and negative dipoles also play an essential role in electrostatic interactions between adjacent chains. The interaction between PETase and the dipoles on the PET’s surfaces may be facilitated by a series of cationic or anionic residues on the enzyme’s surface. These residues are known to establish extensive Coulombic interactions with the ligand’s polar groups. However, ion-pair formation weakens at high salt concentrations due to increased overall entropy, which may contribute to the enhanced catalytic activity of PETase SM14 on PET despite these coulombic interactions.^42^ Furthermore, because it belongs to the marine environment, this microbial hydrolase may exhibit enhanced enzymatic activity in ocean-like conditions. This observation aligns with studies suggesting that salt concentrations (0–1500 mM NaCl) increase PETase activity.^43^ One possible explanation involves the loops in the active site where substrate-interacting residues are arranged. The high ionic force caused by dissolved salts may induce structural changes in this region, altering the spatial arrangement of the ester bond. This reorientation could bring the ester bond closer to the catalytic triad or change its orientation, thereby promoting hydrolysis.

## Conclusions

The accumulation of poly­(ethylene terephthalate) (PET), a highly resistant material with an annual production of 80 million tons, poses significant threats to the environment and health. Bioremediation with purified enzymes represents a promising and environmentally acceptable solution for plastic waste disposal. In this work, a new PETase was successfully produced in E. coli and purified by IMAC chromatography. ESI-ORBITRAP-MS for peptide-mass fingerprinting was performed to confirm the correct amino acid sequence of the enzyme. HPLC analysis of PET degradation products demonstrated the presence of TPA, MHET, and BHET, which are established markers of enzyme activity. PETase SM14 exhibited optimal performance at pH 9.0 and showed thermostability up to 50 °C. After 3 days of incubation, the highest concentration of released TPA was 0.187 mM. Notably, the addition of 900 mM NaCl enhanced enzyme activity by more than 100-fold, likely due to conformational changes in the protein at pH 9.0 and its interactions with the substrate. Additionally, the presence of salt may weaken the substrate’s structure, further facilitating enzyme activity.^41,42^ The crystal structure of PETase SM14 was solved at 1.43 Å resolution, and a comparative analysis with *Is*PETase and PE-H revealed high structural similarity, with RMSDs of 0.691 and 0.81 Å, respectively. Overall, our findings provide novel valuable insight into a polyester hydrolase from a marine source. Due to its thermostability and activity on PCP, PETase SM14 may represent an interesting tool for enzymatic PET waste management.

## Materials and Methods

### PETase SM14 Sequence Analysis

The target protein (*PETase*) was identified in the PAZy database (https://www.pazy.eu/doku.php) among the 119 sequences recognized as acting on PET. The protein sequence spanning residues 25–284, classified by InterPro automatic annotation as a cutinase, is provided in the Supporting Information along with details of the expression vector (Figure S7).

### Production of PETase SM14 in E. coli

The gene encoding the mature PETase sequence from Streptomyces sp. SM14 (UniProt ID: A0A679PDB4) was designed for Ligation Independent Cloning using the aLICatorR system (ThermoFisher) and purchased from IDT. LIC was used to generate the *PETase*-pLATE52 plasmid, which was then used to transform BL21 (DE3) E. coli competent cells. Transformed cells were selected on LB plates containing 100 μg/mL ampicillin. Positive colonies were screened by colony-PCR and grown overnight at 37 °C with shaking at 250 rpm. For protein expression, a 1 L LB/ampicillin culture was grown at 37 °C, 170 rpm, and induced with 0.5 mM isopropyl β-D-1-thiogalactopyranoside for 3 h (Figure S8). Cells were then harvested by centrifugation (10,000 rpm, 10 min), resuspended in 50 mM phosphate buffer pH 8.0 (5 mL/g of biomass), and sonicated on ice using a Microson Ultrasonic cell disruptor. The supernatant was loaded on a HisTrap HP column and purified using the following buffers: buffer A (50 mM phosphate buffer, pH 8.0) and buffer B (50 mM phosphate buffer pH 8.0, 500 mM imidazole). The column was washed with 2 column volumes of buffer A, and the protein was eluted using a linear gradient of buffer B. Peak fractions (Figure S9) were collected. Protein concentration was determined by measuring *A*_280_ and using an extinction coefficient (ε_280_) of 45,380 M^–1^ cm^–1^ and a MW of 31,386 Da, as calculated using the ExPASy ProtParam Tool (https://web.expasy.org/protparam/).

### PETase SM14 Mass Spectrometry

MS/MS analyses were performed using a UHPLC–MS Q Exactive instrument (ThermoFisher). Protein samples were extracted from a single band on the SDS–PAGE. The results were analyzed by MASCOT software (http://mascot.cigs.unimo.it/mascot/) to identify the protein and verify its sequence accuracy.

### Circular Dichroism

CD spectra were recorded on a Jasco J-1500 spectrophotometer at 20 °C. CD spectroscopy was performed to analyze the enzyme’s secondary structure and thermostability. Both experiments were conducted on the enzyme after the His tag removal. Spectra were averaged over three scans using a 2.0 mm quartz cuvette with 2.5 μM samples in a 50 mM Tris (pH 8.0) and 150 mM NaCl buffer. Thermal denaturation curves were recorded in 2.0 mm cuvettes sealed with Parafilm with 2.5 μM samples. Temperature-induced denaturation was monitored at 222 nm, increasing at a rate of 1 °C/min from 5 to 110 °C. Readings were taken at 1 °C intervals with a 1 nm bandwidth and a 10 s response time. Thermal-denaturation midpoints (Tm) were determined by fitting the data to a sigmoidal transition curve using the Boltzmann function. The secondary structure of different variants was measured at 200–250 nm wavelengths at 20 °C. Ellipticity versus wavelength was plotted using GraphPad Prism 10 software, with each spectrum averaged over three scans.

### Thermal Shift Assay

The melting temperature of each variant was measured using differential scanning fluorimetry with Sypro-Orange dye (ThermoFisher Scientific) on an Applied Biosystems 7500 Real-Time PCR system (ThermoFisher Scientific). For each condition, 12.5 μL of enzyme at the starting concentration of 10 μM was added to 12.5 μL of a 10× Sypro-Orange dye solution diluted from a 5000× stock in the same buffer that was used for the protein to reach the final volume of 25 μL, a 5× final concentration of the dye and a 5 μM concentration of the enzyme. All the Tm measurements were performed in 3 replicates (Figures S10–S12). Fluorescence was monitored during the thermal denaturation occurring to the protein upon increasing the temperature from 15 °C to 95.3 °C.

### Crystals of PETase SM14

Crystals of the enzyme without the His-tag were grown at room temperature by using the vapor diffusion method. A 1 μL aliquot of a 10 mg/mL protein sample was mixed with 1 μL of a solution containing 0.2 M ammonium sulfate, 0.1 M MES monohydrate pH 6.5, and 30% w/v polyethylene glycol monomethyl ether 5000. Crystals, which appeared within 1–2 weeks, were frozen in a chemically identical solution supplemented with 25% (v/v) glycerol prior to X-ray diffraction data collection.

### Data Collection and Processing

Diffraction data were obtained using a Eiger2 XE 16M detector and a radiation of wavelength of 0.71326 Å on the I03 beamline at the Diamond Light Source (Oxfordshire, United Kingdom). Data processing was performed using the AutoPROC package. Data collection and refinement statistics are summarized in Table S2.

### Structure Determination and Refinement

For PETase SM14 structure determination, initial data was obtained through molecular replacement using Phaser, with the atomic coordinates of the AlphaFold model (AF-A0A679PDB4-F1) serving as the starting model. Refinement was performed through iterative rounds of manual adjustments in Coot and automated refinement using REFMAC5. Water molecules were added manually and automatically using the refine tool in Coot from the CCP4 cloud package.

### PETase SM14 Activity Assays

The enzymatic activity of PETase SM14 was evaluated using PET PCP, while varying parameters such as temperature and pH. Reaction tubes were set up by adding a squared piece of PCP (*A* = 1 cm^2^) into 400 μL buffer containing 1 μM protein solution. The degrading activity of the enzyme toward post-consumer plastic was tested at different pH values; 100 mM phosphate buffer was used to run tests at pH 6.0 and 7.0, while 100 mM Tris–HCl was used for tests at pH 8.0 and 9.0. The range of NaCl concentration was 0 mM, 300 mM, 500 mM, 700 mM, 900 mM, 1000 mM, 1100 mM, 1300 mM, and 1500 mM. After an incubation time of 72 or 168 h, the reaction tubes containing the PCP were vigorously mixed using a Vortex mixer, and then the substrate was removed with tweezers, washed with SDS 1%, then rinsed with ddH_2_O and finally with 98% ethanol, while the supernatant was filtered and further analyzed by RP-HPLC. For every set of reactions, two control samples were prepared following the same procedure: one was prepared without adding the protein to the reaction mixture, while the other was prepared without adding the substrate.

### RP-HPLC Analyses

The reaction supernatants were dried using a Thermo ScientificTM SavantTM DNA SpeedVacTM Concentrator Kit and resuspended in H_2_O/acetonitrile. Solution A consists of 10% formic acid Milli-Q water, while solution B is acetonitrile. An Agilent Poroshell 120 EC-C18 column was equilibrated with a mobile phase of 80:20 (solution A/solution B) until pressure and UV parameters reached stability. Then, 20 μL of each sample was loaded into the column and eluted over a 20 min run at a flow rate of 1 mL/min at room temperature with the following elution program: 80:20 (solution A/solution B), followed by a 15 min linear gradient 20:50 (solution A/solution B), and 2 min isocratic 50:50 (solution A/solution B). Then, to return to the starting point, 3 min linear gradient from 50:20 (solution A/solution B) and 2 min isocratic 80:20 (solution A/solution B) were applied. The absorbance was measured at 240 and 254 nm. To determine peak areas, the baseline was drawn manually and calculated using the instrument’s software. TPA and BHET were the two main products obtained. Their calibration curves were generated by injecting standard solutions at concentrations of 0.5 2.5 mM, 10 mM, 20 mM, and 30 mM for TPA and 0.05, 0.1, 0.25, and 0.4 mM for BHET. According to Figure S13, the reaction product with the highest retention time was BHET (2.6 min), followed by MHET (2.2 min, assumed) and TPA (1.6 min).

### Scanning Electron Microscopy Imaging

The morphology of PCP films before and after enzyme exposure was examined by SEM on a FEI Nova NanoSEM at an accelerating voltage of 10 kV. Samples were metallized in a Gold Sputter Coater Emitech K550 for 60 s at 18 mA. Digitized images were brought into Epax genesis software for assembly.

### Atomic Force Microscopy

Protein activity was also assessed by analyzing the plastic pieces after incubation with the enzyme by AFM. Morphological characterization of PET samples was performed using an NT-MDT SMENA Solver platform (Moscow, Russia); the analysis was performed in semi-contact mode, and the images were analyzed using Gwyddion 2.67 freeware (http://gwyddion.net).

## Supplementary Material



## Notes

Gene sequence of PETase SM14 reported alongside the expression vector, SDS–PAGE analysis, purification chromatogram, peptide mass spectra of the amino acids sequence determined via MS/MS, protein’s CD spectra, shift in melting temperature in different conditions, thermal shift assay results, HPLC analysis of reaction products with calibration curves, comparative surface electrostatic potential analysis, AFM image of the PCP sample control, and diffraction data collection and refinement statistics (PDF)
The final crystallographic coordinates of the crystal structure of PETase SM14 are available in the RCSB PDB (accession code: 9HYD)

## ⊥

Department of Chemistry, Materials and Chemical Engineering “G. Natta”, Politecnico di Milano, Piazza Leonardo da Vinci 32, 20133 Milano, Italy

## Abbreviations

PET: polyethylene terephthalate
TPA: terephthalic acid
MHET: 2-hydroxyethylterephthalic acid
BHET: bis­(2-hydroxyethyl) terephthalate
RP-HPLC: reverse phase high-performance liquid chromatography
SEM: scanning electron microscopy
AFM: atomic force microscopy
PCP: post-consumer plastic
CD: circular dichroism
Tm: melting temperature
RMSD: root mean square deviation
IMAC: immobilized metal affinity chromatography
MES: 2-(*N*-morpholino) ethanesulfonic acid
ESI-ORBITRAP-MS: electron spray ionization orbitrap mass spectrometry
LCC: leaf-branch compost cutinase
EC: enzyme commission number
isPETase: PETase from Ideonella sakaiensis
PETase SM14: PETase from Streptomyces sp. SM14
PE-H: polyester hydrolase
DSF: differential scanning fluorimetry
TSA: thermal shift assay
