# Supplementary material for: Functional and Structural Characterization of PETase SM14 from Marine-Sponge Streptomyces sp. Active on Polyethylene Terephthalate
Source: ACS Sustain Chem Eng. 2025 May 15;13(20):7460–8. doi: 10.1021/acssuschemeng.5c00737 (PMC12117601; doi:10.1021/acssuschemeng.5c00737)
Supplement: Supplementary file 1 [file sc5c00737_si_001.pdf]

Supporting Information

Functional and Structural characterization of PETase SM14 from marine-sponge Streptomyces sp. active on Polyethylene terephthalate

Alan Carletti[a], Shapla Bhattacharya[b,c], Sara Pedroni[a], Marcello Berto[a], Riccardo Bonettini[a], Rossella Castagna[b,&], Emilio Parisini[b,d], Giulia Di Rocco*[a]

[a]*Department of Life Sciences, University of Modena and Reggio Emilia, Via Campi 103, 41125 Modena, Italy.*

[b]*Department of Biotechnology, Latvian Institute of Organic Synthesis, Aizkraukles 21, LV-1006 Riga, Latvia*

[c]*Faculty of Materials Science and Applied Chemistry, Riga Technical University, Paula Valdena 3, LV-1048 Riga, Latvia*

[d]*Department of Chemistry "G. Ciamician", University of Bologna, Via P. Gobetti 85, 40129 Bologna, Italy*

[&]*Present address: Department of Chemistry, Materials and Chemical Engineering "G. Natta", Politecnico di Milano, Piazza Leonardo da Vinci 32, 20133 Milano, Italy*

*Corresponding author: giulia.dirocco@unimore.it

Contents of the Supporting Information

Total number of pages: 10

Total number of Figures: 13

Total number of Tables: 2

```
  1 AQNPHERGPD PSNSYIEQAR GSYSVSQRSI SRLGSDGFRD GTMYYPTSTA
 51 DGRFGVVAIS PGYTASESTI AWLGPRLASF GFVVVTINTD SRYDQPRQRA
101 TQLHAALDHA IGDSVVGPRI DTSRQAVMGH SMGGGGALQA AEERDEIRAA
151 VPLTPWNLKK GWSGVDAATL VIGAENDAIA PVRSHSIPFY ESLTNAERRA
201 YLELRREGHF APNSSNTLIA KYSVSWLKRY VDNDLRYDQF IDPGPRTGIT
251 TGVSDYRLG
```

Figure S1: MS/MS spectra (100-1200 m/z) obtained from the band of PETase SM14 after purification, extracted and digested with trypsin and analyzed via ESI-MS/MS spectrometry. Sequence coverage of 95%.

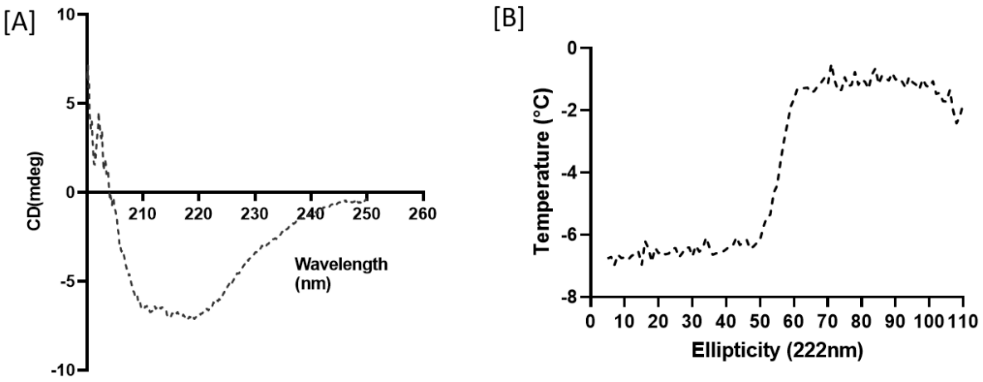

Figure S2. Circular dichroism (CD) analysis to detect the secondary structure of the enzyme (A) and to measure the thermostability of the enzyme (B), as described in the materials and methods section. The determination of the midpoints of the thermal-denaturation curves (Tm) involved fitting the data to a sigmoidal transition curve using the Boltzmann function.

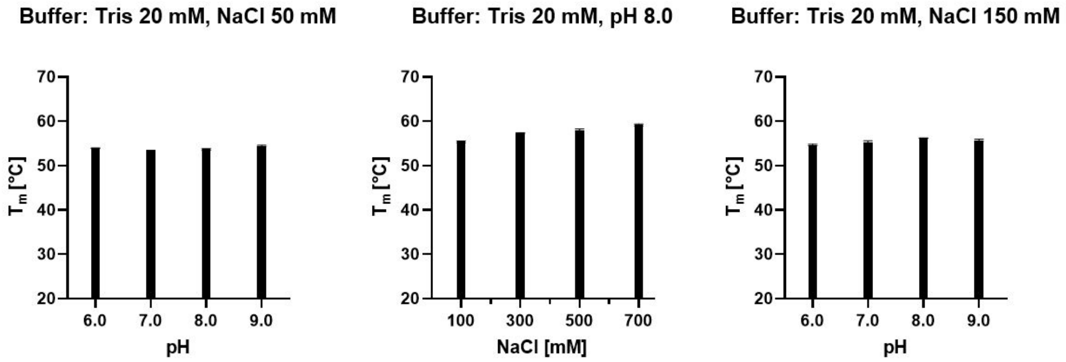

Figure S3. Shift in melting temperature of PETase SM14 in different buffer conditions detected via temperature-dependent fluorescence shifts of SYPRO Orange.

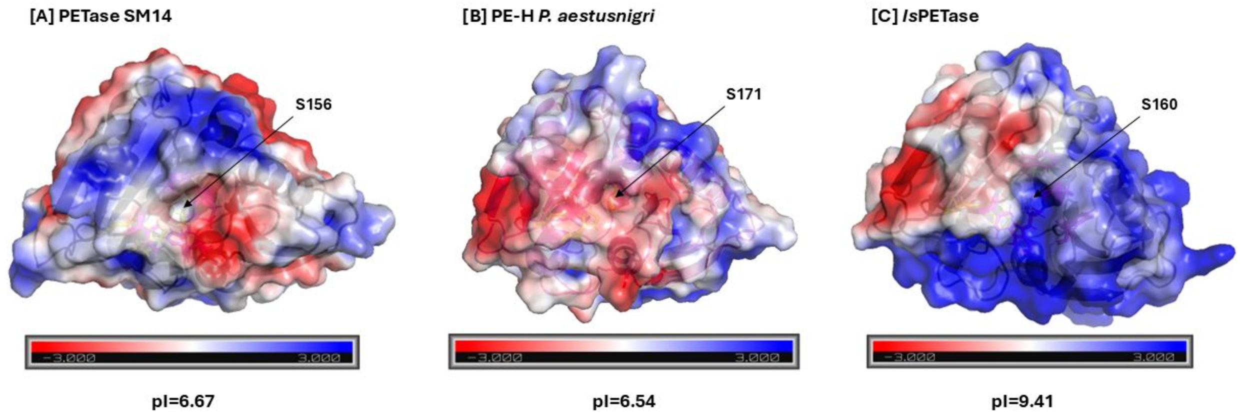

Figure S4: Surface Electrostatic Potential at pH 7.0 of PETase SM14 [A], PE-H from *Pseudomonas aestusnigri* (pdb 6SBN) [B] and *Is*PETase (pdb 6ILW) [C]. The enzyme orientation shows the pocket of the active site from above, with the side chain of the serine of the catalytic triad in the center, indicated by an arrow. As shown by the color legend, red regions (negative potential) result from the clustering of negative charges near the surface, while the blue ones (positive potential) indicate positively charged surfaces. White regions represent neutral potential. To identify areas with strong potential, the low and high range was set to -3 and +3, respectively.

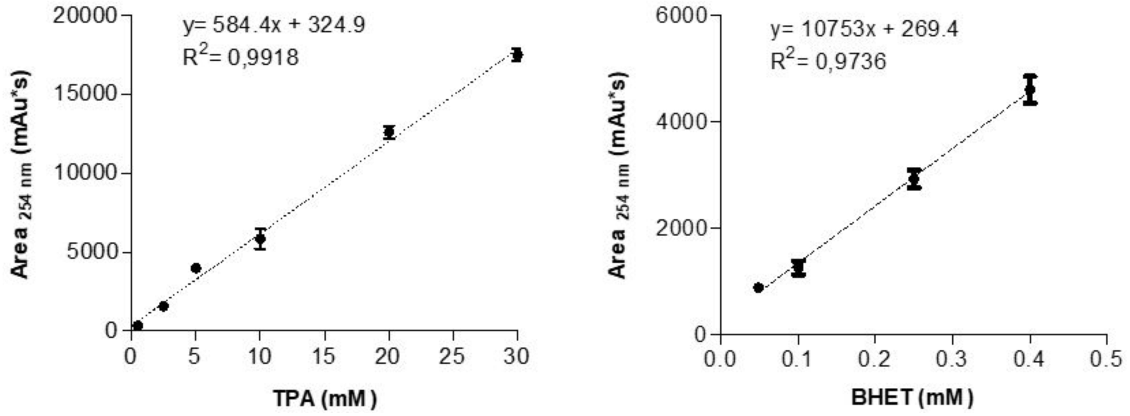

Figure S5. TPA and BHET calibration curves at 254 nm wavelength. (n=3) the error bars when not visible fall within the size of the symbols. The linear regression for TPA is y=584.4 x + 324.9 (r^2 = 0.9918), for BHET is y=10753 x + 269.4 (r^2 = 0.9736).

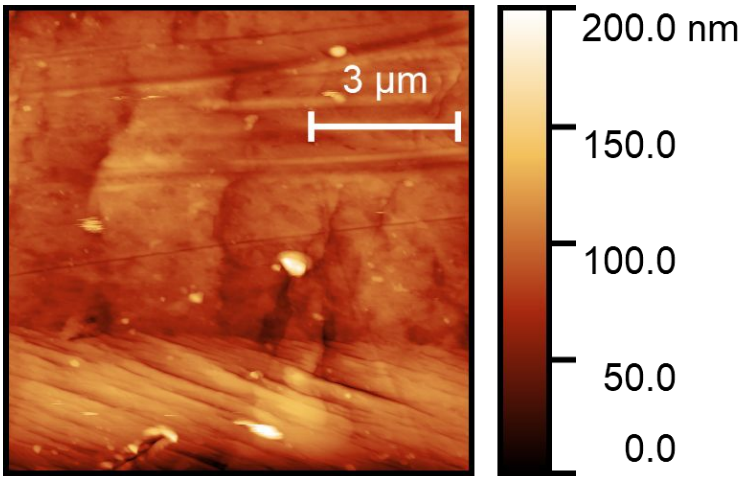

Figure S6. AFM topographical image (10 µm x 10 µm) of PCP sample incubated in 0.9 M NaCl without PETase (control sample).

The sequence of the protein of interest (PETase SM14) without the signal peptide is shown below and it is available on UniProt (ID: A0A679PDB4 · A0A679PDB4_9ACTN). The nucleotide segments in red are complementary to the vector and were added to the protein sequence to allow the lic independent cloning.

GGTTGGGAATTGCAAGCTCAAAACCCCCATGAACGCGGCCCGGATCCATCCAAC
AGCTACATCGAACAAGCGCGCGGAAGTTACAGTGTTAGCCAGCGTAGTATTAGCC
GACTTGGGTCTGATGGGTTTCGTGATGGGACCATGTATTATCCGACTAGCACGGC
AGATGGACGTTTTGGCGTGGTGGCAATCTCTCCGGGCTATACGGCCAGCGAGTCC
ACCATTGCGTGGCTGGGTCCGCGCCTGGCGTCATTTGGTTTTGTGGTTGTGACCAT
TAATACCGACAGTCGCTATGATCAGCCACGGCAGCGTGCGACACAGCTGCACGCC
GCTCTGGACCACGCAATTGGCGACTCGGT TTGACACTTCGC
GTCAGGCAGTAATGGGTCATTCCATGGGAGGTGGTGGTGCCTTGCAGGCAGCGG
AGGAGCGCGATGAAATCCGGGCTGCTGTCCCGTTGACCCCTTGGAACCTCAAAAA
AGGTTGGTCGGGTGTGGATGCGGCGACCCTGGTCATCGGCGCCGAGAATGACGC
CATAGCCCCGGTGCGGTCCCATTCTATCCCGTTCTACGAATCTTTAACAAATGCGG
AACGCCGTGCCTATCTTGAACTGCGTCGAGAAGGCCACTTCGCGCCTAACTCAAG
CAACACGCTGATTGCAAAATACAGCGTCTCATGGTTAAAGAGATACGTTGATAAT
GATCTGCGCTATGATCAATTCATCGACCCGGGCCCACGCACCGGCATTACTACGG
GCGTATCGGATTATAGGCTAGGCTAATGACTTCCCATCTCC.

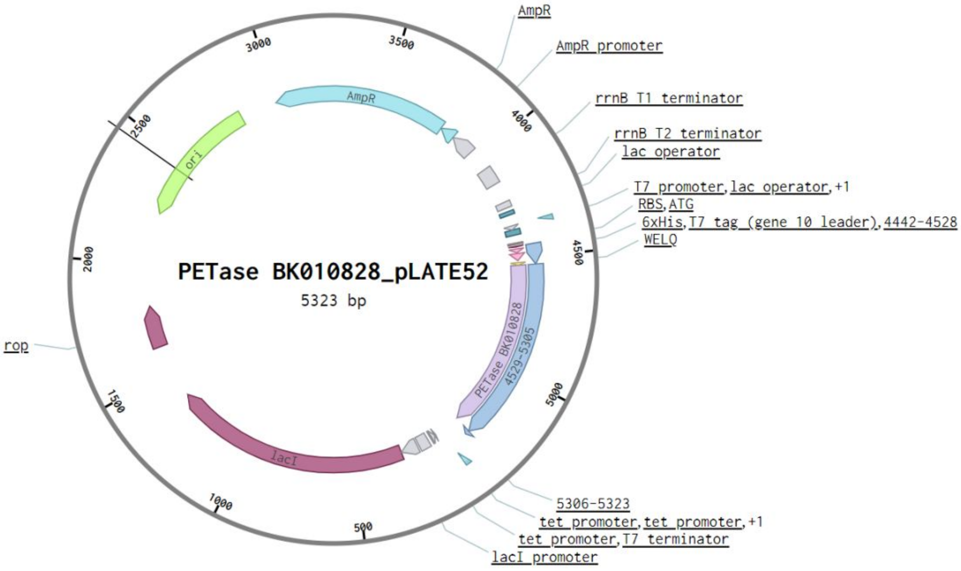

Figure S7. Schematic representation of pLATE52-PETase SM14 (BK010828) vector.

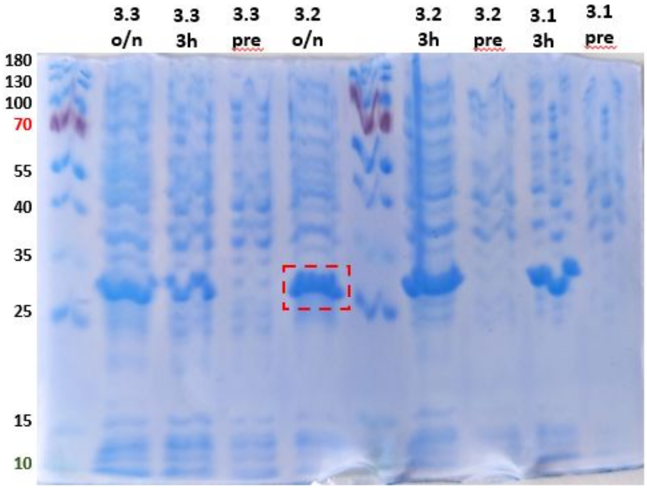

Figure S8. SDS-PAGE of *E. coli* cell extracts from small-scale expression optimization experiments. Samples labelled 3.3 derived from a growth at 25 C°, inducted with 1 mM IPTG. Samples labelled 3.2 derived from a growth at 37°C and induction with 0.5 mM IPTG. Finally, samples labelled 3.1 derived from a growth at 37°C and induction with 1 mM IPTG. In all pre-IPTG samples (pre 3.3, 3.2, and 3.1) no bands around 30kDa were observed, indicating that the repression system of the expression vector was efficient in preventing basal expression. Lowering the temperature to 25°C resulted in reduced protein expression compared to 37°C, as evidenced by the decreased band intensity of the 3.3 samples. Notably, the peak protein concentration in the cells was achieved within 3 hours of incubation following induction of expression.

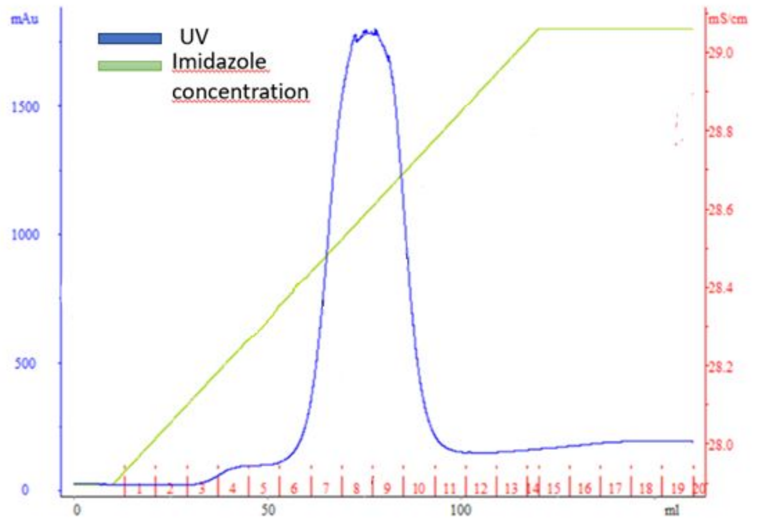

Figure S9. Chromatogram obtained during PETase SM14 purification. IMAC purification of (His)6-tagged PETase SM14 produced in *E. coli.* The tagged PETase SM14 trapped by the His Trap column was eluted in an Imidazole gradient (20 to 500 mM).

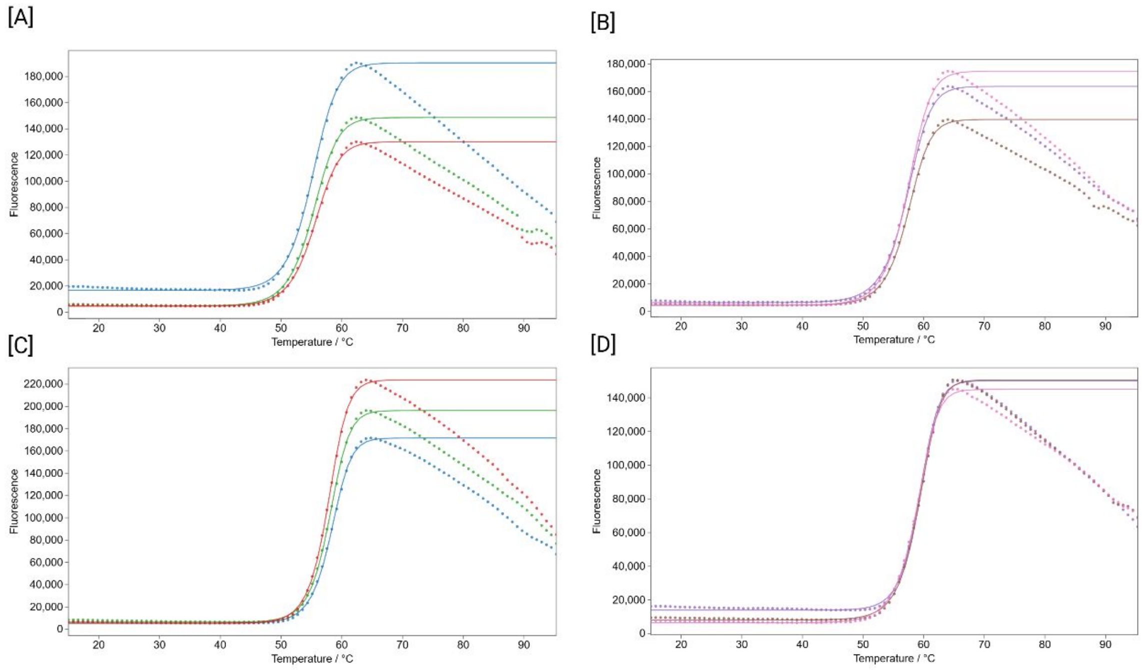

Figure S10. TSA melting plot of PETase SM14 in buffer Tris 20 mM, NaCl 50 mM with different pH [A] pH 6.0, [B] pH 7.0, [C] pH 8.0, [D] pH 9.0.

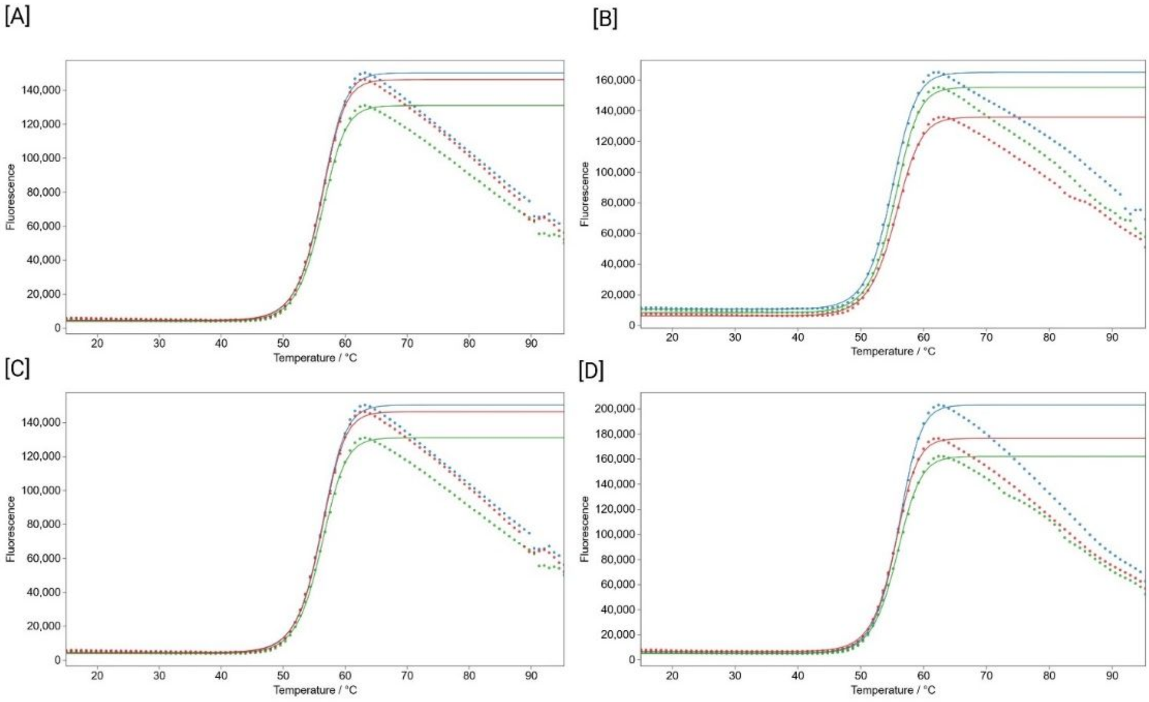

Figure S11. TSA melting plot of PETase SM14 with Tris 20 mM pH 8.0 with different NaCl concentration [A] 100 mM, [B] 300 mM, [C] 500 mM, [D] 700 mM.

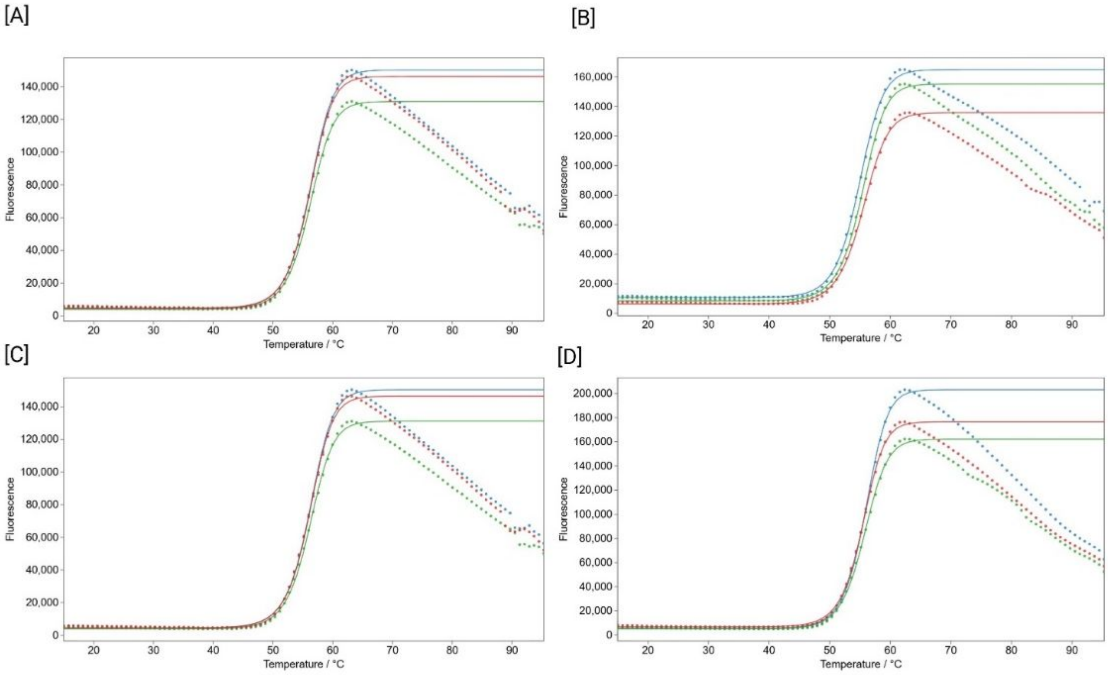

Figure S12. TSA melting plot of PETase SM14 with Tris 20 mM, NaCl 150 mM with different pH [A] pH 6.0, [B] pH 7.0, [C] pH 8.0, [D] pH 9.0.

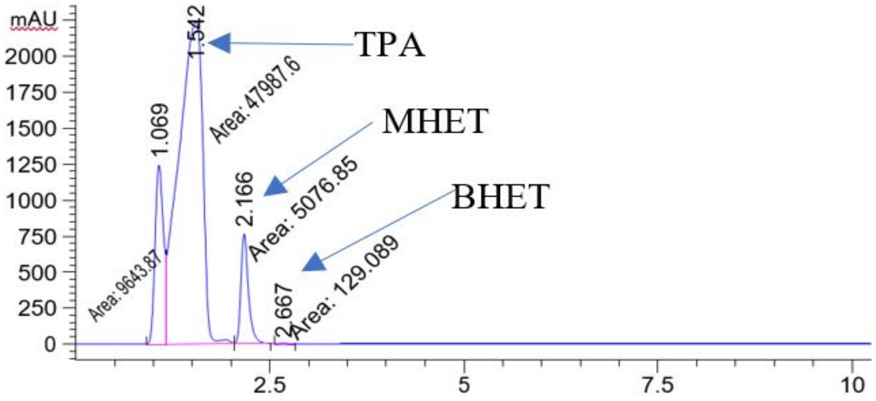

Figure S13. HPLC analysis of the PET hydrolysis products present in the supernatant obtained by the digestion with PETase SM14 on PCP. The separation occurred as described in the materials and methods section. The concentrations of hydrolyzed products (BHET, MHET, and TPA) were detected at 254 nm and calculated from the areas of the adsorption peaks using calibration curves established from TPA and BHET standard solutions. The retention times of TPA and BHET were in the order of 1.6 and 2.6 minutes, respectively. MHET obtained by hydrolyzing BHET standard solution using PETase showed a retention time of 2.2.

**Buffer condition**	**pH**	**T_m [°C]**
**Tris 20 mM, NaCl 50 mM**	6.0	53.93 ± 0.05
	7.0	53.50 ± 0.00
	8.0	53.70 ± 0.16
	9.0	54.43 ± 0.12
Buffer condition	**NaCl Concentration**	**T_m [°C]**
**Tris 20 mM, pH 8.0**	100	55.37 ± 0.17
	300	57.37 ± 0.05
	500	58.03 ± 0.21
	700	59.17 ± 0.19
Buffer condition	**pH**	**T_m [°C]**
**Tris 20 mM, NaCl 150 mM**	6.0	54.70 ± 0.16
	7.0	55.27 ± 0.26
	8.0	56.17 ± 0.05
	9.0	55.73 ± 0.21

Table S1. Melting temperatures T_m [°C] of PETase SM14 with different pH and NaCl concentrations

	PETase SM14
**Wavelength (Å)**	0.71326
**Resolution range (Å)**	83.99 - 1.43 (1.481 - 1.43)
**Space group**	P 6_1
**Unit cell (Å, º)**	96.983 96.983 44.815 90 90 120
**Total reflections**	960914
**Unique reflections**	45062
**Multiplicity**	21.3 (17.6)
**Completeness (%)**	99.92 (99.68)
**Mean I/sigma(I)**	8.9 (0.5)
**Wilson B-factor**	19.65
**R-merge**	0.155 (4.612)
**R-meas**	0.158 (4.750)
**R-pim**	0.034 (1.114)
**CC1/2**	0.999 (0.305)
**Reflections used in refinement**	44589 (4419)
**Reflections used for R-free**	2260 (249)
**R-work**	0.1436 (0.3310)
**R-free**	0.2051 (0.3547)
**Number of non-hydrogen atoms**	2346
**Macromolecules**	2020
**Ligands**	30
**Solvent**	296
**Protein residues**	259
**RMS(bonds) (Å)**	0.010
**RMS(angles) (°)**	1.62
**Ramachandran favored (%)**	97.67
**Ramachandran allowed (%)**	2.33
**Ramachandran outliers (%)**	0.00

**Rotamer outliers (%)**	0.00
**Clashscore**	1.73
**Average B-factor**	28.42
**Macromolecules**	25.87
**Ligands**	50.84
**Solvent**	43.56

Table S2. Diffraction data collection and refinement statistics. Statistics for the highest-resolution shell are shown in parentheses.
